# Role of *PUF60* gene in Verheij syndrome: a case report of the first Chinese Han patient with a de novo pathogenic variant and review of the literature

**DOI:** 10.1186/s12920-018-0421-3

**Published:** 2018-10-23

**Authors:** Qiong Xu, Chun-yang Li, Yi Wang, Hui-ping Li, Bing-bing Wu, Yong-hui Jiang, Xiu Xu

**Affiliations:** 1 Developmental and Behavioral Pediatric Department & Child Health Care Department, Children’s Hospital of Fudan University, 399 Wanyuan Road, Shanghai, 201102 China; 2 Department of Pediatrics, Duke University School of Medicine, Durham, NC 27710 USA; 3 Department of Neurobiology, Duke University School of Medicine, Durham, NC 27710 USA; 4 Program in Genetics and Genomics, Duke University School of Medicine, Durham, NC 27710 USA; 5 Cellular Molecular Biology, Duke University School of Medicine, Durham, NC 27710 USA

**Keywords:** *PUF60*, Verheij syndrome, Intellectual disability, Chinese Han patient

## Abstract

**Background:**

Verheij syndrome is a rare microdeletion syndrome of chromosome 8q24.3 that harbors *PUF60*, *SCRIB*, and *NRBP2* genes. Subsequently, loss of function mutations in *PUF60* have been found in children with clinical features significantly overlapping with Verheij.

**Case presentation:**

Here we present the first Chinese Han patient with a de novo nonsense variant (c.1357C > T, p.Gln453*) in *PUF60* by clinical whole exome sequencing. The 5-year-old boy presents with dysmorphic facial features, intellectual disability, and growth retardation but without apparent cardiac, renal, ocular, and spinal anomalies.

**Conclusions:**

Our finding contributes to the understanding of the genotype and phenotype in *PUF60* related disorder.

## Background

Verheij syndrome (VRJS) (MIM 615583) is characterized by intellectual disability, growth retardation, dysmorphic facial features, and vertebral skeletal abnormalities. Additional features include coloboma and renal and cardiac defects [1–5]. Verheij syndrome is caused by a deletion in chromosome 8q24.3. The commonly deleted intervals include two genes, poly(U)-binding-splicing factor (*PUF60*) and scribbled planar cell polarity protein (*SCRIB*). Point mutations in *PUF60* have been reported in individuals with clinical features overlapping with those associated with a 8q24.3 deletion or VRJS [2]. The *PUF60* gene encodes a protein that directly interacts with splicing factor 3B, subunit 4 (SF3B4) and plays a role in the recognition of the 3′ splice site and the recruitment of U2 and U5 small nucleolar ribonucleoprotein to the intron for splicing [2]. Mutations of the *PUF60* gene including nonsense, frameshifting, splicing site, or missense have been identified in 24 individuals [1, 2, 4–9]. Here, we report a Chinese Han patient who carries a heterozygous de novo and novel nonsense mutation (c.1357C > T, p.Gln453*) in *PUF60* identified by clinical whole exome sequencing. This individual shares some characteristic features with previously described individuals including intellectual disability, growth retardation, and dysmorphic facial features but not other features such as cardiac, renal, ocular, and spine abnormalities [1, 2, 4–9]. This is the first report and characterization of a Chinese Han child harboring a *PUF60* variant.

## Clinical summary

**Fig. 1:**
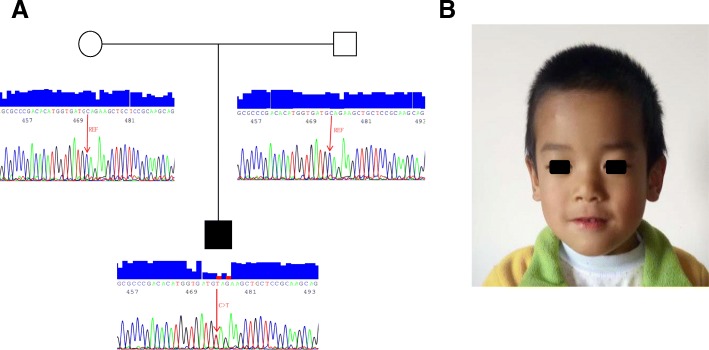
A patient with a de novo heterozygous de novo PUF60 variant. **a** Sanger sequencing confirmation for c.1357C > T PUF60 variant in proband but absence in both parents. **b** A facial profile to patient. Noted for short neck, thin upper lip, long philtrum, micrognathia and wide nasal bridge, and narrow almond-shaped palpebral fissures

A 5-year-old boy was born prematurely at 36 weeks of gestation without a known cause. The birthweight was 2650 g and appropriate for gestational age. He had a significant history of poor feeding and failure of thrive as an infant. At the age of 4 years and 7 months, his weight was 13.5 kg (< 2 SD) and his height was 98 cm (< 2 SD). His head circumference was 49.5 cm (< 1 SD). He showed global developmental delay and started to walk at the age of 20 months. At the age of 4 years, his vocabulary was limited to just a few words. At present, he is able to speak simple phrases but not complex sentences. In addition, he has significant history of chronic diarrhea from birth to 2.5 years of age without an identifiable cause. He had an average of 5–7 loose stools per day while being breastfed during the first year. Between the ages of 1–2.5 years, he was formula-fed and had an average of 6–8 loose stools per day. The chronic diarrhea resolved after 2.5 years of age without any clinical intervention. He was also diagnosed with febrile seizures and had significant sleep disturbance. Upon the physical examination at AGE, a distinct facial dimorphism was noted and these include a short neck, thin upper lip, long philtrum, wide nasal bridge, micrognathia, and almond-shaped eyes and short palpebral fissures (Fig. 1). There was no ocular coloboma and no shoulder subluxation or generalized joint laxity. Both parents were healthy and non-consanguineous. Family history was negative for any neurodevelopmental disorder or known genetic disease. Endocrine work-up, brain magnetic resonance imaging, abdominal and renal ultrasonography, and skeletal bone survey were normal. His hearing was normal. The detail clinical description of this patient and comparison with other previously reported cases carrying *PUF60* mutations are listed in Table 1.

**Table 1:** Comparison of clinical features in our patient and others previously reported with the *PUF60* mutation

Clinical phenotypes	Patient 1	Previous reported with *PUF60* variants (*n* = 24)^[2, 4–9]^	Previous reported with 8q24.3 deletion (*n* = 7)^[1, 2]^
Gestation			
Pre-term	+	3/18	NA
Full-term		15/18	1/1
Height (z score < 2 SD)	+	16/23	7/7
Renal	–	6/22	4/7
Coloboma	–	8/23	4/7
Cardiac	–	13/21	5/7
Skeletal	–	15/23	5/7
Hand anomalies	–	11/20	4/7
Joint laxity	–	11/19	5/7
Feeding	+	10/17	5/7
ID (intellectual disability)	+	24/24	5/6
Auditory	–	8/14	1/5
Hypertrichosis	–	5/12	NA
Facial feature			
Long philtrum	+	16/23	7/7
Thin upper lip	+	15/23	7/7
Micro-retrognathism	+	13/22	4/7
Short neck	+	14/22	5/7
Wide nasal bridge	+	9/22	6/7

## Genetic evaluation

Standard karyotyping was normal (46, XY)*.* A clinical trio whole exome sequencing (WES) was performed by WuXi NextCODE Genomics, Shanghai, China (CLIA Lab ID: 99D2064856) using a previous described protocol [10]. Briefly, exome capture was performed using the Agilent SureSelect Human All Exon V5, Illumina TruSeq Rapid PE Cluster, and SBS kits (Agilent Technologies, Santa Clara, CA, USA). WES was performed on the Illumina HiSeq 2000/2500 platform. Reads were aligned to the human genome reference sequence (GRCh37/hg19 build of UCSC Genome Browser; http://genome.ucsc.edu) with the Burrows-Wheeler Aligner v.0.6.2. Duplicate paired-end reads were marked with Picard v.1.55 (https://broadinstitute.github.io/picard/). The Genome Analysis Toolkit v.2.3–9 was used for base quality score recalibration, indel realignment, and variant discovery. Variants were annotated using a pipeline developed in-house [10] and filtered in the Exome Variant Server, gnomAD, Exome Aggregation Consortium, or the dbSNP databases. The candidate variants were confirmed by Sanger sequencing.

**Fig. 2:**
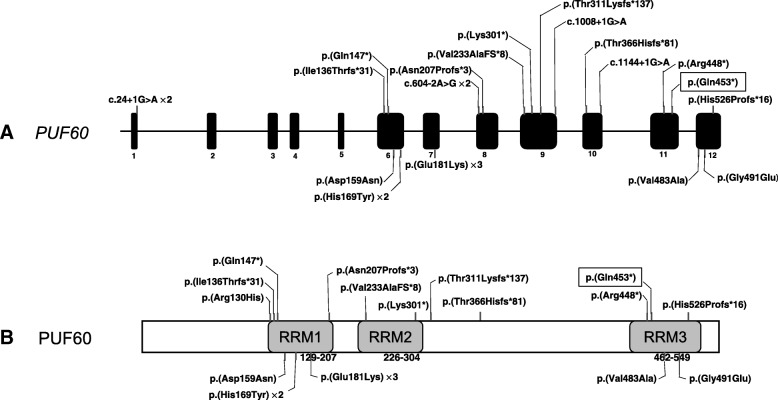
Genetic location of the PUF60 variants identified to date. **a** Variants identified in PUF60 which are loss of function variants (stop codon, splicing and frameshift mutations) on top and missense variants below the gene. The variant reported in this case showed with square frame. We used bar to report the variants and “×” represents the number of cases. The size of exon and intron is not proportional. **b** Distribution of amino acid changes related to the protein domains RRM, RNA recognition motif in PUF60 protein

In this proband, a heterozygous de novo and nonsense c.1357C > T variant in exon 11 (NM_078480.2) was identified from the WES analysis and confirmed by Sanger sequencing. This variant is predicted to result in a premature stop codon (p.Gln453*) of PUF60 protein (Fig. 1). Other previously reported pathogenic variants are also diagramed in Fig. 2 for a comparison.

## Discussion and conclusion

In this report, we presented the finding of a novel pathogenic variant in *PUF60* gene in a Chinese child. To our knowledge, this is the first case of a Chinese child with *PUF60* mutation. The proband’s clinical presentations of intellectual disability, short stature, and dysmorphic facial features were similar with those previously reported cases with mutations in *PUF60* variants or with a deletion of 8q24.3 containing the *PUF60* or VRJS [1, 2, 4–9]. However, the vertebral anomaly, coloboma, renal defects, and cardiac defects reported in other cases were not found in our patient. In individuals with 8q24.3 deletion or VRJS, both *PUF60* and *SCRIB* genes are deleted. In an early study in zebrafish, morpholino-mediated knockdown of either *PUF60* or *Scribble* (*Scrib*) in zebrafish recapitulates some of the phenotypes of 8q24.3 deletion in humans [2]. Knockdown of either gene cause a short stature, microcephaly, and reduced jaw size. Knockdown of *Scrib* alone resulted in coloboma and renal abnormalities, whereas knockdown of *PUF60* alone resulted in cardiac structural defects. Knockdown of both genes result in more severe short stature phenotype. It was concluded that *PUF60* or *SCRIB* haploinsufficiency drives the majority of syndromic phenotypes found in patients with the copy number deletion of 8q24.3 or VRJS. However, several patients harboring *PUF60* point mutations have been recently described, and these individuals have clinical phenotypes that overlap significantly with patients carrying 8q24.3 deletions. These findings support a major role for *PUF60* in the phenotype of VRJS in human [1, 2, 4–9]. It remains to be seen whether mutations in *SCRIB* alone in humans may also cause the clinical problem similar to mutations of PUF60. The presentation of chronic diarrhea in our patient has not been previously reported [1, 2, 4–9]. It will be interesting to see if diarrhea is a feature of other patients harboring a *PUF60* defect.

The findings of a heterozygous de novo nonsense change of *PUF60* in our study further supports haploinsufficiency as the underlying mechanism [6]. Loss-of-function variants are predicted to result in altered dosages of different PUF60 isoforms and, consequently, abnormal splicing of targeted genes [2]. Both VRSJ syndrome and *PUF60*-related disorder encompass diverse phenotypes, suggesting that dysregulated targeted genes due to the *PUF60* deficiency may account for diverse phenotypes in humans.

Interesting to note, two reports have suggested the overlapping clinical features between *PUF60* related disorder with CHARGE syndrome. In these cases, the clinical and genetic evaluation of CHARGE syndrome is considered but the mutation study of *CHD7* is negative (PMID 29300383 and PMID: 28471317). Other have also suggested the overlapping feature between Cornelia de Lange syndrome and other craniofacial disorders caused by mutations in genes encoding the spliceosomal proteins [11, 12]. The apparent question for the future investigation is whether there is a convergent molecular mechanism among these disorders.

The majority of genetic variants in *PUF60* are predicted to be loss of function mutations, However, missense variants are also reported. Clinically, there is no significant difference between individuals carrying clear loss of function and missense variants. These may support the loss of function mechanism underlying the missense variants in these cases. However, additional phenotypical and molecular studies are warranted to clarify the genotype and phenotype correlation and whether the missense variants may result in loss of function at protein level.

This study is the first report of a Chinese Han patient carrying de novo *PUF60* heterozygous mutation. The proband exhibited many of the characteristics previously reported in PUF60 variant or VRSJ patients such as intellectual disability, growth retardation, and dysmorphic facial features [1, 2, 4–9]. However, the patient did not present with especially vertebral skeletal abnormalities, coloboma, renal defects, or cardiac defects. Clinical and molecular characterization of patients with diverse background will help us better understand the genetic diversity and prevalence of *PUF60* related disorder.

## Abbreviations

PUF60: Poly(U)-binding-splicing factor
SCRIB: Scribbled planar cell polarity protein
SF3B4: Splicing factor 3B, subunit 4
